# Population Intervention Effects of Spatial Social Polarization on Dementia Disparities

**DOI:** 10.1093/geroni/igaf122.947

**Published:** 2025-12-31

**Authors:** Hoda Abdel Magid, Mengya Xu, Wei-Lin Wang, Evelyn Gonzalez, Jennifer Ailshire, Julie Zissimopoulos

**Affiliations:** University of Southern California, Los Angeles, California, United States; University of Southern California, Los Angeles, California, United States; University of Southern California, Los Angeles, California, United States; University of Southern California, Los Angeles, California, United States; University of Southern California, Los Angeles, California, United States; University of Southern California, Los Angeles, California, United States

## Abstract

Spatial social polarization (SSP), the unequal geographic distribution of privilege and deprivation, may contribute to disparities in dementia prevalence and incidence. This study used the Index of Concentration at the Extremes (ICE) to measure SSP across five domains: race, income, education, primary language, and homeownership. Using a 20% sample of 2019 Medicare claims data (N > 4 million), we applied Population Intervention Models (PIMs) with the parametric g-formula to estimate dementia outcomes under hypothetical scenarios where SSP disparities were eliminated. These counterfactual simulations compared current exposure distributions to idealized scenarios, such as universal privilege or universal deprivation. Dementia prevalence was 8%, with incidence at 3.5%, disproportionately affecting Black populations. In the hypothetical scenario where all individuals lived in high-income neighborhoods, dementia incidence was projected to decrease by 0.4%, whereas a scenario where all individuals lived in income-deprived neighborhoods was projected to increase incidence by 0.5%. In a scenario where all individuals lived in predominantly White neighborhoods, dementia incidence was projected to decrease by 0.1%, whereas residence in predominantly Black neighborhoods was projected to increase incidence by 0.3%. Similarly, a scenario where all individuals lived in neighborhoods with higher educational attainment was associated with a projected 0.4% reduction in dementia incidence, while lower educational attainment corresponded to a projected 0.8% increase in prevalence. These findings illustrate potential population-level impacts of SSP disparities under counterfactual scenarios and provide insight into how neighborhood factors may shape dementia risk.

